# Patient Centric Pharmaceutical Drug Product Design—The Impact on Medication Adherence

**DOI:** 10.3390/pharmaceutics12010044

**Published:** 2020-01-03

**Authors:** Enrica Menditto, Valentina Orlando, Giuseppe De Rosa, Paola Minghetti, Umberto Maria Musazzi, Caitriona Cahir, Marta Kurczewska-Michalak, Przemysław Kardas, Elísio Costa, José Manuel Sousa Lobo, Isabel F Almeida

**Affiliations:** 1 CIRFF, Centre of Pharmacoeconomics, Department of Pharmacy, University of Naples Federico II, Corso Umberto I, 40, 80138 Napoli NA, Italy; enrica.menditto@unina.it (E.M.); valentina.orlando@unina.it (V.O.); 2 Department of Pharmacy, University of Naples Federico II Corso Umberto I, 40, 80138 Napoli NA, Italy; gderosa@unina.it; 3 Department of Pharmaceutical Sciences, Università degli Studi di Milano, Via G. Colombo 71, 20133 Milan, Italy; paola.minghetti@unimi.it (P.M.); umberto.musazzi@unimi.it (U.M.M.); 4 Division of Population Health Sciences, Royal College of Surgeons, Beaux Lane House, Mercer Street, Dublin 2, Ireland; caitrionacahir@rcsi.ie; 5 Department of Family Medicine, Medical University of Lodz, 60, Narutowicza St., 90-136 Lodz, Poland; m.kurczewska@o2.pl (M.K.-M.); przemyslaw.kardas@umed.lodz.pl (P.K.); 6 UCIBIO/REQUIMTE, Faculty of Pharmacy and Porto4Ageing, University of Porto, Rua Jorge Viterbo Ferreira, 228, 4050-313 Porto, Portugal; emcosta@ff.up.pt; 7 UCIBIO/REQUIMTE, MedTech-Laboratory of Pharmaceutical Technology, Department of Drug Sciences, Faculty of Pharmacy, University of Porto, Rua Jorge Viterbo Ferreira, 228, 4050-313 Porto, Portugal; slobo@ff.up.pt

**Keywords:** adherence, fixed dose combinations, patient centric pharmaceutical drug product design, 3D-printing

## Abstract

Medication adherence is a growing concern for public health and poor adherence to therapy has been associated with poor health outcomes and higher costs for patients. Interventions for improving adherence need to consider the characteristics of the individual therapeutic regimens according to the needs of the patients. In particular, geriatric and paediatric populations as well as dermatological patients have special needs/preferences that should be considered when designing drug products. Patient Centric Drug Product Pharmaceutical Design (PCDPD) offers the opportunity to meet the needs and preferences of patients. Packaging, orodispersible formulations, fixed dose combinations products, multiparticulate formulations, topical formulations and 3D printing are of particular relevance in a PCDPD process. These will be addressed in this review as well as their impact on medication adherence.

## 1. Introduction

### 1.1. Taxonomy

During the last four decades of adherence research, different definitions of the process of deviating from recommended treatment have been employed. The terms “adherence” and “compliance” have been used synonymously in the literature; however, the term adherence has gained popularity in recent years as it implies a more mutual and dynamic interaction between patients and health care providers and emphasizes the importance of medication-taking behaviour. While, the use of the term compliance, which implies that the patient has a passive role in the process of medication taking, has diminished [1]. Due to this heterogeneity in the definitions being used across scientific literature, it was necessary to obtain a consensus on the terminology and develop a taxonomy of adherence. The ABC project (Ascertaining Barriers to Compliance) has recently developed an adherence taxonomy which provides concise and adequate definitions to serve the needs of both clinical research and medical practice [1,2].

**Figure 1:**
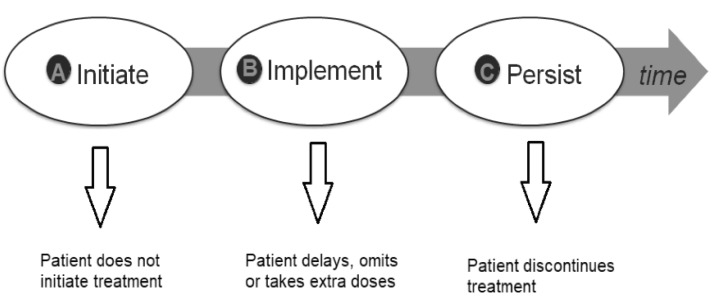
Ascertaining Barriers to Compliance (ABC) taxonomy of medication adherence describing its three key steps. Adapted of reference [1].

Medication adherence has been defined as an active, cooperative and voluntary participation of the patient in following recommendations from a healthcare provider [1]. This multifactorial behaviour encompasses three critical steps (Figure 1):

- Initiation, which defines the moment that the patient takes the first dose;
- Implementation, which is the extent to which a patient’s actual dosing corresponds to the prescribed dosing regimen, from initiation until the last dose;
- Persistence, which is the length of time between initiation and the last dose.

Moreover, the ABC taxonomy clearly distinguishes between procedures that describe actions through routines that have been established (medication adherence and management of adherence) and the sciences that address those procedures (adherence-related sciences) [2]. The terms concordance and persistence have also been used to define some patients’ medication-taking behaviours. Concordance implies that the patient and the healthcare professionals came to an agreement about the treatment that the patient should follow, acknowledging that they may have different points of view, while persistence relates to the time interval between the first and the last dose of medicine prescribed [1,2,3,4]

### 1.2. Impact on Public Health

Adherence to medication is widely recognized as an increasingly relevant issue to healthcare systems as poor adherence to therapy is associated with negative health outcomes and higher patient costs. Unfortunately, this aspect is broadly underestimated in real world practice and by patients [5].

Adherence to therapy is especially important for chronic diseases’ management, diseases also known as noncommunicable diseases (NCDs). As defined by the World Health Organization (WHO), NCDs involves “health problems that require continuous treatment over a period of time from years to decades.” Approximately 80% of people over 65 suffer from heart failure, respiratory failure, sleep disorders, diabetes, obesity, depression, dementia, hypertension, which often occur simultaneously in the same individual [6]. Cardiovascular diseases account for most NCDs deaths, (17.9 million people annually), followed by cancers (9.0 million), respiratory diseases (3.9 million) and diabetes (1.6 million) [7]. In 2020, NCDs will represent about 80% of all diseases in the world, for which about 70–80% of resources will be committed globally. 

Several studies have shown that adherence to chronic therapies is far from expected, with approximately 50% of patients not taking their medications as prescribed [8,9,10,11]. A recent cross-country European study showed that the proportion of treatment discontinuation for NCDs was 55.63% for antihyperlipidemics, 60.24% for antiosteoporotics and 46.80% for oral antidiabetics [8], within a year. The prevalence of non-adherence in patients suffering from NCDs is higher in developing countries given the scarcity of health resources and inequalities in access to health care. The patient population at greatest risk of nonadherence is older patients with polytherapy [12]. Multimorbidity is increasingly present among older populations, increasing the complexity of the therapeutic regimen for all stakeholders and ultimately impacting negatively on health outcomes [13,14]. Furthermore, there is an alarming lack of evidence about treatment of patients with multiple concurrent chronic diseases [15,16]. 

Interesting results are reported in a recent analysis conducted by the IMS (Institute for Health Care Informatics), which estimated the economic impact of the use of inappropriate drugs in 186 countries [17]. The study considered six NCDs of high social impact such as diabetes, osteoporosis, heart failure, HIV, hyperlipidaemia, hypertension, estimating at approximately 300 billion dollars cost of using non-optimal drug therapies. It showed that two thirds of these costs are attributable to approximately ten million avoidable hospitalizations, equivalent to about 140 billion dollars. In particular, the issue causing highest cost was nonadherence to therapy, with a value of almost 50% of the total. The WHO estimates that the cost of nonadherence to drug therapy amounts to 125 million euros per year in Europe, including costs from avoidable hospitalizations, nursing home admissions and premature deaths. A study by Sokol et al., 2005, related to four NCDs including diabetes, showed that a high level of adherence to therapy in diabetes is associated with lower costs related to illness and lower costs of hospitalization [18]. Although adherence can lead to increased pharmacy costs, total health care cost is reduced due to reductions in hospitalization rates’ [19]. Hospitalization is the largest component of medical costs, so it is likely that the changes in hospitalization risk are the primary driver of the cost savings observed at higher levels of adherence.

### 1.3. Determinants of Non-Adherence

Multiple factors can influence low levels of adherence to medications. Based on WHO recommendations, the causes of non-adherence are classified into five main dimensions: ‘*socioeconomic factors, health care and system-related factors, therapy-related factors, condition-related factors and patient-related factors’* [20,21,22]. A review of systematic reviews by Kardas et al. identified and classified the determinants of patient adherence per the ABC Adherence Taxonomy using the WHO 5 factor model. The review found that socioeconomic factors, such as family and social support, employment status, cost of drugs and/or treatment influence adherence. Furthermore, although non-adherence has often been perceived as the fault of patients, there was evidence that healthcare system factors, such as access to healthcare, unclear information about drug administration, as well as poor provider-patient relationship may have an important impact on adherence. Low adherence was also related to the condition and the asymptomatic nature of the disease, as well as disease severity [23]. Therapy-related factors, such as the complexity of the therapeutic regimen, duration, past failures, therapeutic changes or adverse events and factors related to the patient such as forgetfulness, beliefs, knowledge and/or skills inadequate to the management of therapy and misunderstanding instructions for treatment, were also found to strongly impact on adherence [24]. Another systematic review identified almost 800 individual factors, among which frequent dosing, a high number of prescribed medications, drug formulation or poor taste, were found to be related to the implementation component of adherence behaviour using the WHO 5 factor model [23,25]. Therapy-related factors, such as patient (un) friendly regimes have also been shown to influence older patients’ beliefs and concerns about their treatment, which in turn indirectly influences their adherence [26]. Patient concerns about their medication have been shown to increase with the extent to which the medications interfere in conducting day to day activities or social events, leading to non-adherence [27]. 

Research to date indicates that medication non-adherence is affected by multiple determinants and many of these determinants are related to each other. Moreover, it is increasingly evident that adherence, as a process and the determinants of adherence have to be assessed over time and not just at one evaluation time point (drug discontinuation) [28,29]. To develop effective interventions to increase medication adherence it is important to focus on determinants that are modifiable and can be identified and targeted for change. Interventions for the improvement of therapeutic adherence should not only focus on the clinical aspects of the treatment but consider the characteristics of the individual therapeutic regimens according to the needs of the patients, in order to be effective. Many of the adverse health outcomes associated with non-adherence may in fact be preventable, if measures are taken to address the therapy-related determinants of non-adherence and optimize medication-taking ability.

## 2. Patient Centric Pharmaceutical Drug Product Design: Defining Target Product Profiles for Special Populations 

Patient centric pharmaceutical drug product design (PCDPD) can be defined as the “process of identifying the comprehensive needs of individuals or the target patient population and utilizing the identified needs to design pharmaceutical drug products that provide the best overall benefit to risk profile for that target patient population over the intended duration of treatment” [30]. The patient centric drug product design process starts with a target drug product and a patient profile considering the patients’ context. Understanding patient preferences and needs regarding treatment features is a key element of the patient centric design process. The different patient factors (Table 1) should be considered and prioritized to achieve a more universal design, suitable for the broadest patient population [31].

Drug product in the context of patient centric design is considered the presentation of the treatment to the end-user (patient/care giver/health care provider) which includes the dosage form, formulation, dose, dosing frequency, packaging (primary, secondary and tertiary), medical device, dosing devices and instructions for use (Table 2). Although increased development costs can arise in the implementation of PCDPD, the involvement in the early stages of the design process of pharmaceutical technologists with good fundamental knowledge on dosage forms and development and practical experience on industrial-scale manufacturing can represent an essential contribution to the constrain of costs.

Integrating these characteristics in the design process helps achieving a drug product adapted to the needs and preferences of a target patient population. Some populations present particular complexity with respect to medication adherence and will be addressed below.

### 2.1. Elderly

The life expectancy in developed countries has grown in the last decades, increasing the proportion of older people with respect to the total population. In addition, the development of novel pharmacological treatments has reduced disease mortality and increased the number of illnesses that can be controlled by chronic therapies. Also, there has been a shift from inpatient to outpatient settings across various areas, for example, neoplastic diseases. As a consequence, the proportion of older patients that receive more than one medicinal product each day has significantly increased with respect to the past.

This demographic change has pushed manufacturers to design and produce medicinal products based on the biopharmaceutical features of the elderly [33]. Indeed, their health is generally affected by a gradual decline of motor function (e.g., dysphagia, trembling hands, reduced flexibility), cognition (e.g., impaired cognition, dementia) and sensory acuity (e.g., impaired vision, hearing loss). The efficacy and safety profiles of a medicinal product can be altered in the elderly population with respect to adults. Both pharmacokinetics and pharmacodynamics of an active pharmaceutical ingredient (API) are affected by ageing. For example, the distribution and the metabolism of an API can be prejudiced by reduced renal and hepatic functions. Its biodistribution can be altered by the changes in the ratio of human body surface area to body weight and the percentage of fatty mass. On the other side, the use of specific excipients (e.g., phosphate salts) can overload the renal function in older patients who have already been compromised [34]. 

Despite these peculiarities, the elderly have not been generally recruited, contrary to younger adults, into the clinical studies carried out to obtain a marketing authorization for medicinal products. To overcome this limitation, European Medicines Agency (EMA) released in 1994 the guideline “ICH E7–studies in support of special populations: geriatrics,” in which the agency stressed, for the first time, the need to recruit patients aged 65 years or older into the clinical trials. In the following years, the EMA released additional guidance to applicants promoting more accurate classification criteria of the recruited patients, clinical endpoints and tools that can be used to characterize the physical frailty of the older patient [35,36]. 

Since the decline of motor and sensory function of the elderly, an increased risk of medication errors and poorer adherence to the pharmacological therapies are observable in the real world due to difficulties in swallowing and handling the drug products [33,37]. As a consequence, several aspects have to be considered in the quality target product profile of a drug product intended to be administered in older people [38]. 

**Figure 2:**
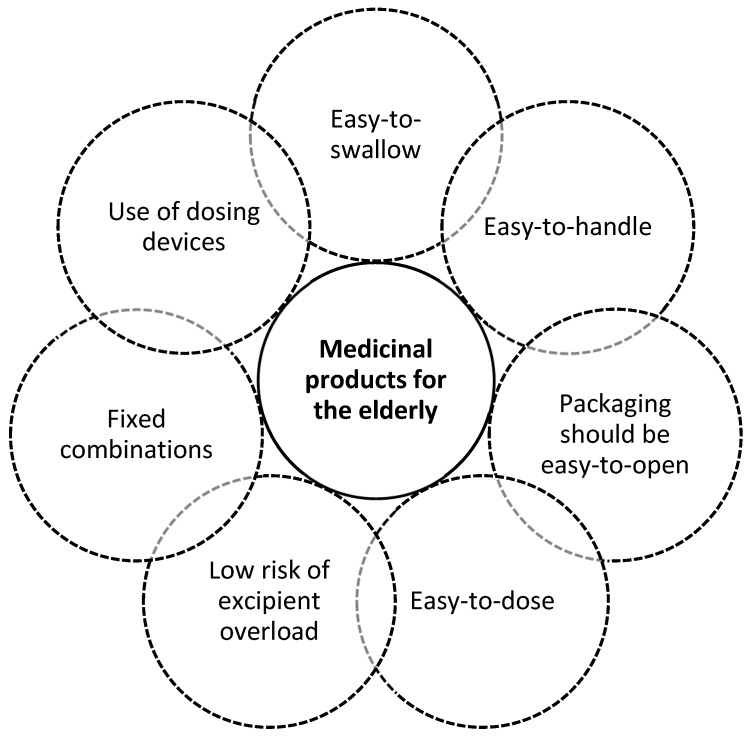
Target product profile of medicinal product intended to be used by the elderly.

As reported in Figure 2, the pharmaceutical development of an elderly-centric drug product should be based on a holistic approach. This should include not only attributes with a strong impact on the biopharmaceutical performances of the drug product but also those with an influence on the adherence of the patients, such as the handling and dosing. In this light, the use of multi-compartment medication devices has been demonstrated to be effective in supporting older patients in their daily regimens and in improving clinical outcomes [39,40]. Indeed, such tools permit the patient/caregiver to organize in advance the medicines to take in a specific day period (e.g., morning, evening) or day of the week, reducing the risk of medication errors and simplifying the actions that patients have to do for medicine administration. A proper design of the packaging, labelling and the leaflet information are also important aspects to be considered in the development of a patient centric drug product for the elderly. The design, the fonts and the other graphical aspects of both the packaging and the leaflet should be designed to be readable by a patient with impaired vision in order to facilitate the adherence and reduce the risk of misuse or medication errors [41]. For example, it should be preferred not coloured standard fonts, high dark/light contrast and point sizes higher than 12 [42]. Moreover, the leaflet should include specific information for the elderly (e.g., drug interaction information in the older population).

Finally, the development of fixed dose combinations (FDCs) seems desirable, especially for multimorbid patients, in order to reduce the medicines that have to be taken by the patients and to improve adherence to pharmacological therapies [43].

### 2.2. Paediatric Population

Paediatric patients are another special population that needs patient centric medicinal products. As well as geriatrics, the majority of medicinal products available on the markets have not been indicated for paediatric patients since the clinical trials performed in the pre-authorization stage of the product generally do not include children. This lack of information is due to several causes including ethical issues related to testing on children. 

In 2007, the European Union released a Regulation to assure a better benefit/risk balance for medicinal products with paediatric indications and to increase the availability of age-appropriate and paediatric-centric medicines on the market [44]. Among the innovations introduced by Regulation No 1901/2006, it is noteworthy the upgrade of regulatory requirements to obtain the marketing authorization with a paediatric indication. In particular, applicants should provide a paediatric investigation plan (PIP), which should include details of the timing and measures proposed to demonstrate the quality, safety and efficacy of a medicinal product in paediatric populations. In the following years, the EMA released several guidelines to support applicants in the pharmaceutical development [45] and in the clinical trials of paediatric medicinal products [46,47]. The following aspects should be considered to define the quality target product profile of a paediatric medicine: patient age and its developmental physiology, the peculiar characteristics of the diseases to be treated in the children, the route of administration, the dosing regimen, the maximum duration of the therapy, the age-associated activities of children (e.g., school, nursery), the environment or setting where the product is likely to be used (e.g., hospital or community), the caregivers’ characteristics and their behaviour. All these aspects should be considered in order to assure good patient acceptability.

The scenario is even more complicated considering that many diseases are peculiar of childhood. For example, febrile seizure affects only children ranging for 6 months to five years old. Bronchiolitis is a typical virus infection of the small airways in the lungs that occurs in children less than two years of age. The otitis is frequent in children since children have a different anatomical conformation of the eustachian tube compared to adults facilitating bacterial infections [48].

Therefore, the features of a paediatric-centric drug product change as a function of the age of the patient and, the anatomical, functional, physiological developmental stage of body. Indeed, infant body anatomy and physiology are significantly different from those of children, adolescent (<18 years) or adults. For example, the ability to swallow foods and medicines in infants is lower than in children and adolescents [49]. In this context, one of the most relevant key aspects to be considered in pharmaceutical development is the selection of proper dosage forms based on the target population age [50]. Oral liquid dosage forms with a low dosing volume and rapidly dissolving mini-tablets (diameter 2 mm) can be preferable in infants, whereas immediate release mini-tablets with a diameter of 2 mm, oral powders, granules (administered with soft or semisolid food) can be administered to young children from 6 months. Mini-tablets with 4-mm diameter are suitable for children older than 1 year. Since young children cannot swallow conventional-sized tablets intact until 3 or 6 years, immediate and modified released tablets are not considered suitable for such patient populations. On the contrary, tablets and capsules were better accepted than other solid oral dosage forms by school children and adolescents, due to faster ingestion [51]. However, small tablet diameter (<10 mm) or capsule size (<Size #0) are better preferred by school children than adolescents. Organoleptic properties, such as product appearance, smell and texture may also affect adherence [52]. In this light, the visual appearance of the product should be pleasant enough for the young patient but not too attractive to promote the misuse of medicinal products. As well, the taste and mouthfeel of the product should be optimized for the paediatric population to find a balance between the masking of the unpleasant taste of an API and a low attractiveness of the product to avoid accidental intake because of the patient exchanges the medicinal product as a candy [53,54].

Besides the dosage form, the API salts and excipients should also be carefully evaluated during pharmaceutical development. The API salts should be selected to assure an acceptable bioavailability considering the physiological developmental stage of the patient. As an example, the solubility of API in liquid dosage forms should be enhanced to avoid the administration of high dose volume. For excipients, their risk assessment of the paediatric exposure should be reconsidered. Indeed, the safety profile of an excipient in children cannot be extrapolated tout court from adults due to the different physiological developmental stage and the possible immaturity of the children organ and body systems [45]. Consequently, safe excipients in adults can be toxic to infants or children or cause sensitization or allergies. For example, the use of preservatives in paediatric medicinal products should be carefully evaluated. Some studies have demonstrated that parabens (e.g., propyl paraben) may interfere with the physiological development of reproductive organs in animal models. As a consequence, wherever possible such excipients should be avoided for paediatric formulations. As well, the use of benzyl alcohol in preparations intended to be administered to children up to 3 years old should be carefully evaluated and, preferentially avoided, due to their immature metabolism [55]. In general, age-associated safe levels of exposure are available for many of them. If robust exposure data do not exist, a conservative approach has been promoted by the EMA to preserve the health of young patients.

**Figure 3:**
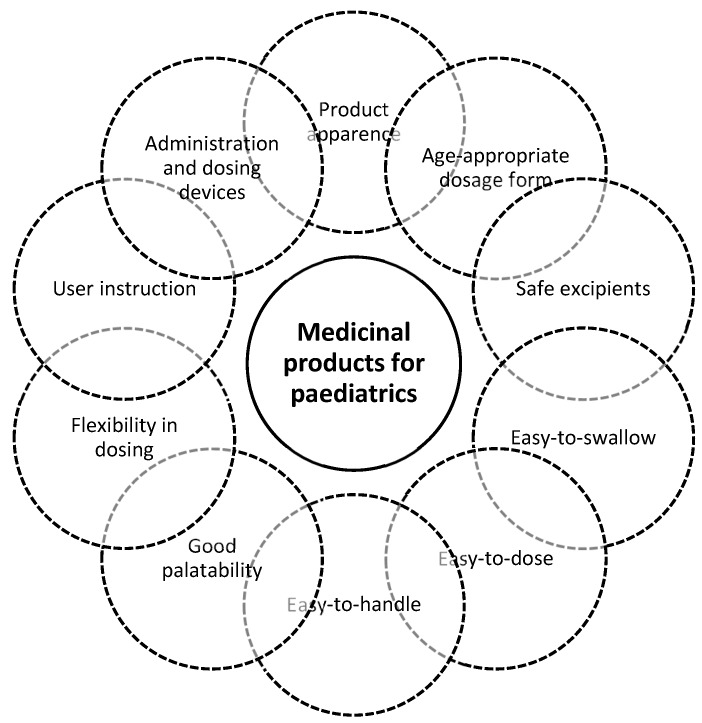
Target product profile of medicinal product intended to be used by paediatric patients.

As outlined above, the dose definition (including the use of specific administration/dosing devices) and its frequency are other aspects that can impact on the acceptability of a medicinal product. In outpatient paediatrics, the dosing errors are frequent in children weighing less than 35 kg or in children under 4 years [56]. In the majority of the cases, the dose for children under 4 years should be adapted according to the patient weight. In this context, if the caregiver is not properly trained, the risk of medicines’ overdoses or underdoses increases. Therefore, the availability of dosing devices and clear instructions for caregivers is important to facilitate patient adherence and avoid medication errors. Taken together all the above-mentioned aspects, a target product profile can be proposed for paediatric drug products (Figure 3).

When a paediatric dose of the drug product is not available on the market, the only possibility to treat children is to tailor the dose by slitting, breaking or crushing a tablet or a capsule (e.g., clobazam capsule for seizures, propranolol tablet for infantile hemangiomas) [57]. In many cases, these procedures are off-label treatments, since it is frequent that the starting medicinal product has not been tested and authorized to be used in children. Such procedure can result in the administration of inaccurate dosing, as well as losing specific benefits of certain dosage forms (e.g., slow release tablets) [57]. To minimize these errors, the design of a proper device on the basis of the shape and size of medicinal products is essential to help caregivers in dosing the medicinal products to assure the reproducibility of dose assumed [58]. Another possible strategy to overcome the unavailability of industrial medicinal product or strength for the paediatric population is the compounding of patient-specific extemporaneous preparation in a pharmacy setting [59]. In this context, the quality, safety and efficacy of the compounded preparations have been assured by the cooperation between physicians and community/hospital pharmacists. In particular, the product quality is assured by the pharmacist professionalism and by procedures to assess the risks connected with the compounding of the preparation based on its target profile. In some cases, health authorities of European Countries, released monographs for specific paediatric preparations (e.g., British Pharmacopeia, Czech National Formulary) to support the prescribing and compounding activities of physicians and pharmacists when industrial products are not available. In this context, strong cooperation between pharmaceutical technologists and regulatory authorities is important to design and develop standardized formulations with suitable biotechnological performance and physicochemical stability to improve the quality of the compounded preparation. 

Finally, the regulatory agencies states that all the information and use instructions should be provided to patients in a clear way to assure correct and full administration of the medicinal product and to minimize the risk of medication errors [45,56]. On one side, the labelling design and the readability of the leaflet are key aspects to be considered to support young patients, especially in the case of over-the-counter products and caregiver in the correct use of the medicinal products. The inclusion of cartoons in the leaflet can be advantageous to improve the comprehension of patient/caregiver regarding the administration procedures (e.g., how to split a tablet or to dose a syrup). On the other side, if administration/dosing devices have to be used, healthcare professionals should train properly the patients and their families to avoid dosing errors.

### 2.3. Dermatological Patients

Dermatological diseases are very common [60]. Different treatment modalities are available and topical treatments are frequently used. Adherence is typically low for topical treatments [61,62]. Patient, treatment and disease factors have been appointed to justify poor adherence. Regarding treatment, poor acceptability of the topical vehicles and staining of clothes have been reported by patients with chronic skin disorders as barriers to optimal treatment adherence. For the particular case of topical medicines, some patient needs can be identified. For instance, patients with impairment of motoric function (e.g., rheumatoid arthritis) might be unable to open closure systems, squeeze the tubes, rub the formulations onto the skin or reach less accessible areas. When skin barrier is damaged, such as in atopic dermatitis or highly inflamed, as in severe psoriasis, avoiding friction deserves special attention. 

**Figure 4:**
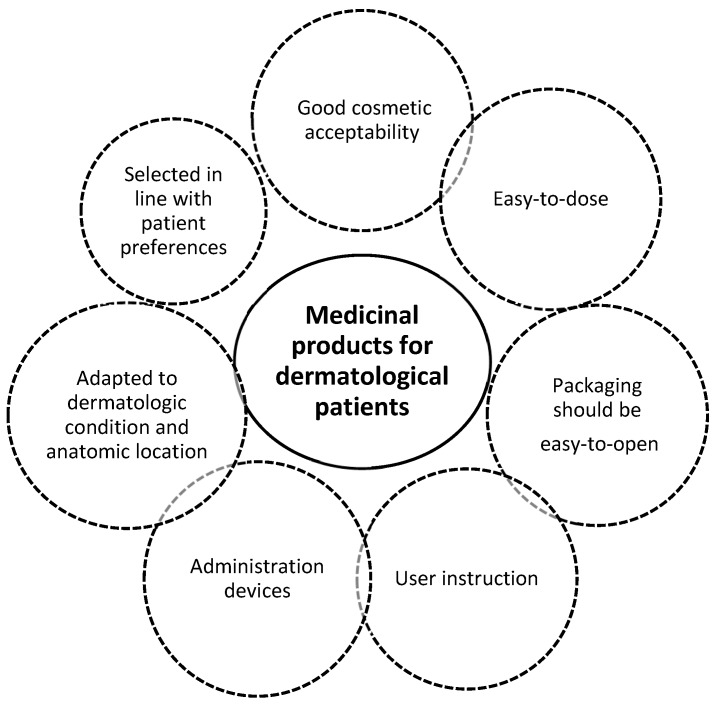
Target product profile of medicinal products intended to be used by dermatological patients.

Patient preferences also vary according to the dermatologic condition being treated [63] and anatomic location of lesions. The physical form has been shown to influence patient preference. For example, acne patients tend to prefer washes, creams and lotions; patients with atopic dermatitis prefer creams; and psoriasis patients prefer creams, ointments and foams (particularly for the scalp) [63]. Specific attributes of topical products were also analysed for psoriasis patients with the three attributes more valued by patients being—to allow dressing shortly after application, good moisturizing properties and use only once daily [64]. These data, if confirmed in a large population, can be largely useful to guide PCDPD. When administration/dosing devices are associated with the drug product, user instructions should be clear and healthcare professionals should educate the patients and their caregivers about their correct use. A standard target product profile can be proposed for medicinal products for dermatological patients (Figure 4).

## 3. Patient Centric Pharmaceutical Drug Product Design: The Impact on Medication Adherence

Translating patients’ preferences and needs into the design of a drug product is the cornerstone of PCDPD. A recent literature review identified only 45 studies focusing on patients’ preferences for pharmaceutical preparations [65]. Of these, only 35 investigated dosage form design and 11 exclusively assessed dosage forms for the oral route. Noteworthy, very few studies have examined patient’s preferences for shape, size and colour of solid dosage forms [66,67], which are very commonly used for the treatment of NCDs. Besides conventional solid dosage forms such as tablets, a wide spectrum of dosage forms can be designed and manufactured by the pharmaceutical industry.

When implementing a PCDP design some drug products emerge more frequently as better adapted to promote adherence. These will be addressed with more detail focusing on their impact on medication adherence.

### 3.1. Packaging

Several packages have been designed to meet patient needs, like helping to remember when to take the medication or assist reading the labels, for instance by using braille inscription. Dose dispensing devices (e.g., inhalers) can be also included in the packages to ease the administration. For topical treatments, applicators that ease the application, avoiding using hands are already on the market (sometimes called “no mess applicators”). Drummond et al. have reviewed preferences for packaging design and found that wing top and screw cap openings, push-through blisters and suppositories with a slide system were favoured. Child-resistant containers were considered difficult to handle by specific patient populations [65].

Different packaging options have been designed with the aim to promote adherence including multidose dispensing systems (MDDS) [68], calendar packaging [69], pill boxes (also called multi-compartment containers/multi-compartment compliance aids-MCAs), blister packaging (prepared by professionals) and bubble packages [70]. 

However, their impact on adherence is still poorly understood. Meta-analysis findings from a systematic review support the use of packaging interventions (pill boxes or blister packs) to effectively increase medication adherence with a 71% adherence rate reported in the treatment group compared to a 63% adherence rate in the control group [40]. A few reports have also emphasized a positive effect on adherence of multi-dose dispensing systems (MDDS) [71] calendar packaging [69] and bubble packages [70]. Even though more studies are clearly needed in this field, these results collectively suggest that packaging interventions could be helpful as part of a combination strategy for adherence promotion, especially for polymedicated patients.

### 3.2. Fixed Dose Combinations (FDCs)

Fixed combination medicinal products offer the opportunity to simplify drug administration. Fixed dose combination (FDC) has been developed to reduce the pill burden for patients. By reducing overall pill burden and simplifying medication regimens, fixed combinations have been shown to improve medication adherence and persistence in several studies [32,43,72,73]. European regulatory authorities consider fixed combinations as “a particular technical or technological processing of therapeutically active substances in order to allow it to be administered to the patient in the simplest, safest and most effective way” [74]. Along with lower pill burden, FDCs often lower the costs of treatment, due to the use of inexpensive, generic compounds [75]. Despite this, FDCs do not follow a simplified procedure for marketing authorization. If the combination of the APIs is well-established for a specific therapeutic indication in the clinical practice, preclinical and clinical studies required for the marketing authorization to assess the combination safety and efficacy can be significantly reduced. In general, bioequivalence studies between the fixed combination products and the single drug products are accepted if no specific pharmacokinetics or pharmacodynamics interaction are expected. Alternatively, the marketing authorization holders of a fixed combination medicinal product have to provide the preclinical and clinical data to support the therapeutic indication. If the fixed combination contains approved APIs not approved as combination therapy or one or more New Active Substances, detailed preclinical and clinical data have to be provided [74]. 

Today, numerous FDCs exist on the market and are widely recognized as safe and effective [43,73,76]. For this reason, many chronic conditions can benefit from this kind of therapeutic approach to reduce pill burden, such as dyslipidaemia, atherosclerosis, hypertension, osteoporosis, heart failure, post myocardial infarction, angina, type 2 diabetes, chronic obstructive respiratory disease and HIV (Table 3).

Recently, FDCs have achieved a key role in management of hypertension due to the fact that most patients need more than two drugs to keep their blood pressure under control, as shown both in clinical trials and in real practice [77]. In 2018, European Society of Hypertension (ESH) and European Society of Cardiology (ESC) published guidelines for the management of hypertension [78]. In this document is stated that therapy with FDCs can be considered in both first and second-line therapies for the attainment of the recommended value of <140/90 mmHg for blood pressure.

Antihypertensive combination drug therapy offers advantages over single drug therapy, partly due to the different sites of actions of each drug and partly due to a lower risk of adverse events. This combination therapy is also important for patients to take their medications properly and continue to take them in long-term treatment. This approach proved to be effective also cost-wise. Indeed, Sherrill et al., in a comparative meta-analysis on health care costs of hypertension management, showed that yearly all-cause total costs were reduced by approximately 2039 dollars in the fixed combination group vs free combination and there was a reduction of about 709 dollars in hypertension and cardiovascular-related costs [79]. 

Any drug from the following classes is proposed for four kind of two-drug FDCs: angiotensin converting enzyme inhibitors and calcium channel blockers, angiotensin converting enzyme inhibitors and diuretics, angiotensin receptor blocker and calcium channel blockers and angiotensin receptor blocker and diuretics. Moreover, also the combinations of angiotensin converting enzyme inhibitors with beta-blockers and calcium channel blockers with diuretics, are available (Table 3). 

It is now about 15 years since the concept of the polypill (defined as the combination usually of 3, 4 or more drugs in single tablet or capsule) has been proposed to further encourage adherence to the treatment of NCDs, in particular cardiovascular diseases [80]. On this basis, triple fixed combinations have been developed for hypertension treatment (i.e., valsartan, amlodipine and hydrochlorothiazide) and for the human HIV type 1 infection [81] (Table 3). The newest therapeutic approach is represented by a quadruple fixed dose combination—the so-called quadpill—which has been approved for hypertension (i.e., irbesartan, amlodipine, hydrochlorothiazide and atenolol) and HIV virus (i.e., emcitarabine, tenofovir, elvitegravir and cobicistat) treatment [82,83,84]. Further examination of the quadpill concept is needed to investigate effectiveness against usual treatment options and longer term tolerability [85].

Some issues should be carefully taken into account when designing and developing formulations based on FDCs. First of all, the combination of drugs in one formulation hamper the tuning of the dose, making a patient centric approach more difficult. Moreover, the design of the drug delivery form must consider biopharmaceutical issues, as well as the pharmacokinetics, of each active substance, sometimes requiring a controlled release. In addition, for each active substance, chemical and physical stability must be assured, also considering the compatibility with the formulation technologies as well as with the used excipients. Indeed, a formulation procedure/pharmaceutical form suitable for a specific active substance can result disadvantageous for another. Finally, interaction among active substances and eventual physical-chemical incompatibility should also be investigated. These issues generally result also in higher development and manufacturing costs. Different technological approaches have been successfully employed for manufacturing FDCs. Among them, the most intuitive approach is the coencapsulation of different solid drugs into hard gelatin capsules [86]. This approach can be also performed extemporaneously in clinical practice by pharmacists. For marketed FDCs, other technological approaches used to prepare oral formulations with a single active substance, such as direct compression, dry granulation, spray drying and wet granulation to create tablets, powders and capsules, have been used [87]. In this case, chemical-physical incompatibility between the co-encapsulated actives must be evaluated during the formulation development. Alternatively, the multilayer tablet technology address incompatibilities of actives by compressing granules of two or more actives as different layers in one tablet. In the case of incompatible actives, layers of non-functional placebo have been used to avoid the interaction between actives present into the other layer [8]. It is worthy of note that, in this technology, the solid state of the active is a mandatory requisite. In this context, an innovative technology named UnigelTM, which is able to coencapsulate different dosage forms in soft capsules, has been developed. This technology allows overcoming incompatibility among components and is suitable for actives at solid, liquid and semisolid state, also maintaining the benefits of soft gelatin capsules.

Furthermore, there is the risk that the final dose form becomes too sizeable, impeding oral administration [88]. This is particularly true for subjects suffering from dysphagia or any other condition that makes difficult to swallow tablets. These are common conditions in the geriatric and paediatric population. This could be solved via the formulation of multi-particulate, oral fast dissolving dosage forms [89] or 3D printing technology, which will be addressed with more detail in this review. Finally, the co-crystallization of drugs can lead to formation of amorphous complexes with solubility characteristics different respect than the naked drug [90]. Thus, different dissolution rate of co-precipitated drugs should be taken into account when designing FDCs, for example, for oral administration.

Several studies showed that FDCs can improve medication adherence by about 25% thanks to the reduction of pill burden and the consequential simplification of medication management [72].

### 3.3. Orodispersible Dosage Forms

Another strategy that can be adopted to improve adherence to therapeutics is the design of delivery systems able to overcome the limitation of conventional oral liquid and solid dosage forms. Liquids (i.e., syrups, suspensions and solutions) are very flexible in dosing and can be easily swallowed by the patient. However, the dose accuracy is strongly affected by the type of measuring device used by the patient (e.g., spoon, syringe) and the risk of misdosing is high due to incorrect handling [91]. Therefore, tablets and capsules are the most commonly used dosage forms. However, they are more difficult to swallow by children and older adults, especially when their shape and size have not been properly optimized. 

To solve these criticisms and improve patients’ adherence, orodispersible dosage forms (ODx) can be a valid technological solution [92]. An ODx is defined as a dosage form intended to be placed in the mouth where they rapidly liberate the loaded API producing a fine suspension or solution in the saliva that can be easy-to-swallow by the patient. 

By a technological point of view, two different classes of ODx are available on the market as medicinal products: orodispersible tablets (ODT) and orodispersible films (ODF). Similar to conventional tablets in organoleptic properties, ODT are designed to disintegrate in less than three minutes after the contact with saliva [93]. Specific production technologies are used to permit the fast disintegration of the ODT—freeze-drying (e.g., Zydis^®^), moulding or direct compression (e.g., Nurofen FlashTab^®^) of specific excipients are some of the technologies applied in the industrial production of ODT. The ODF (e.g., Zuplenz^®^, Risperidon HEXAL SF, Rabestrom^®^) is thin strips of plasticized hydrocolloids or blends that are obtained by a solvent casting technique or hot melt extrusion [92]. Such technologies allow producing in industrial-scale laminates that are properly cut in diverse shapes to obtain the different strengths of the same formulation. The formulative space of ODF (<200 mg) is limited by the reduced size (e.g., 2 × 3 cm^2^) and thickness (<350 µm). In this context, the selection of ODF matrix components results very critical to the production of laminate with proper mechanical properties [94,95].

Both ODT and ODF require specific types of machinery (e.g., freeze drier, solvent casting) and packaging (e.g., moisture protecting packaging) to be produced. Consequently, the production costs of ODT/ODF are higher than conventional tablets and capsules and, therefore, their economic sustainability may be acceptable to market only for specific API or when a significant advantage in terms of bioavailability or patient adherence is expected. 

Contrary to what is expected from fast dissolving dosage forms, the use of an ODx instead of a conventional immediate release dosage form does not affect generally the bioavailability of the API. Indeed, the resistance time in the buccal cavity and oesophagus of the API suspension/solution is too short to sustain a significant drug permeation through the mucosae. Only in the case of selegiline, a significant improvement of its bioavailability was reported [96]. 

Since ODx are easy-to-swallow without drinking or chewing, they do not require water for the medicine’s administration and, therefore, they are better accepted by travellers and bedridden or non-cooperative patients. Their use is also related to a reduced likelihood of suffocation or choking. Moreover, they are well-accepted by who is not able to take or swallow tablets or capsules. Swallowing problems can be due to psychological (e.g., fear), physiological (e.g., dysphagia) and iatrogenic causes [92]. Difficulties in swallowing medicines are particularly relevant in children, older adults [91] and patients with mental health disorders due to a variety of causes such as psychiatric medication side effects or comorbid neurologic conditions [97]. They strongly impact on the adherence of patients [37]. Consequently, the advantages of ODx as patient centric pharmaceutical drug products are mostly due to the high acceptability by the patient. Indeed, the patient’s adherence is generally improved with respect to conventional solid dosage forms [92,98]. For example, the olanzapine ODx show a comparable efficacy and safety profile with respect to conventional solid dosage forms but they are superior in terms of adherence and patient preference [99,100].

However, ODx acceptability is strongly influenced by the taste sensation after the dissolution of the dosage forms in the mouth [53]. Indeed, a proper strategy to taste-making is needed to cover the unpleasant taste of API. However, it is not an easy task due to the small formulative space of ODx and the existing differences in the taste preference among children, adult and geriatric populations [89]. Moreover, the use of moisture protective packaging that cannot be easy-to-open by older people can also limit the acceptability of ODx. Unlike ODT, the ODF may be difficult to handle by older patients with poor manual dexterity due to their shape and their thickness [91].

### 3.4. Multi-Particulate Formulations

Multi-particulate dosage forms are multiple unit systems of mini-tablets or pellets that are filled into capsules, compressed into tablets or used individually. By offering great design flexibility by combining units with different drugs and/or with different release profiles, multi-particulate formulations are good candidates for personalized treatments. Adaptation of the dose, like the administration of an amount of pellets based on body weight, is a clear advantage for the paediatric population. Multi-particulate formulations are also useful for the design of FDCs since individual dosage units containing different drugs can be combined in the final product [101]. Despite the promise hold by multi-particulate formulations these dosage forms are not yet a well-established platform mainly due to manufacture and administration constrains, such as weight control, content uniformity and handling by patients. The impact on adherence has not been yet studied even though higher acceptability and capacity to swallow mini-tablets over syrup has been demonstrated in the paediatric population [102,103].

### 3.5. Topical Formulations

The range of topical vehicles available for the treatment of the different skin diseases is quite large and is presented in distinct physical forms, mainly liquid and semisolid. These can be hydrophilic, lipophilic or bi-phasic products. Topical vehicles can vary substantially regarding mechanical and sensory properties which can impact their use by patients and satisfaction with treatment [104]. Innovations in topical treatments are linked not only to the discovery of new drugs but also to the reformulation of vehicles of already-in use APIs (such as diclofenac salts and the association of calcipotriol and betamethasone) in order to improve administration, bioavailability, ease of use and adherence. Topical vehicles, like foams, emulgels and oleogels, are some examples [105] of what is, at present, available on the pharmaceuticals market. Satisfaction with topical treatment has been already associated with improved adherence [106] which justifies the investment in the drug product design. The impact of the topical vehicle on adherence has been, however, seldom investigated. In clinical practice, one possible way to meet patients’ preferences for topical vehicles is to allow them to try vehicle samples before establishing the treatment plan. Pharmaceutical compounding can also be an important strategy to obtain individualized medicines that are not available on the market. From the industrial point of view, patients’ preferences have to be considered and prioritized to achieve a universal drug product for dermatological patients.

### 3.6. 3D Printing

In the last decade, the applications of 3D-printing to the pharmaceutical field have risen and different technological approaches are available in the literature for preparing medicines tailored to the clinical needs of the patient. With respect to previously discussed patient centric pharmaceutical dosage forms, the 3D-printing is more versatile and flexible in dosing than API and provides personalized treatment to a single patient according to his/her needs. Indeed, most of the investigated printing technologies can be applied not only at the industrial-scale level but also in a pharmacy setting or, potentially, at a patient’s home. In this optic, the most promising technologies are those based on a binder jetting (e.g., inkjet printing) or the material extrusion [e.g., fusion deposition modelling (FDM); semisolid extrusion (SSE), hot melt ram extrusion (HMRE)] [107,108,109].

Such technologies have been suitable for preparing FDCs [110,111,112,113] or to load nanosystems able to modify the release kinetics of the drug substance [114]. For example, Khaled and coworkers provide a proof-of-concept of a 3D-printed FDC dosage form containing up to five APIs with different release profiles (i.e., acetylsalicylic acid, atenolol, hydrochlorothiazide, pravastatin, ramipril).

Inkjet printing is based on the deposition of the corrected dose of API on an edible substrate as solution or suspension. The substrate, which is generally made of water-soluble polymers (e.g., polysaccharides, cellulose derivatives), can be easily cut and handled by the patient [107,115]. Moreover, the inkjet printing can be useful to prepare innovative medicinal products that combine the drug-loaded dosage forms and all the information needed by the patient for proper use. For an example, modulating the deposition design of drug-loaded fluid, Edinger and coworkers were able to prepare ODF in which the drug was printed as quick-response (QR) code that encodes for information relevant for the patient and the health professionals regarding the medicinal product [116]. Since the QR code can be read by a common smartphone, such an approach may be a valid strategy to prevent misusing or medication errors in the future. However, several parameters can impact on the quality of drug product made by inkjet 3D printing. For an example, the solubility/dispersibility of the drug in the fluid, the fluid viscosity, the porosity and the mechanical properties of the edible substrate, other than their possible interaction and compatibility are only a few aspects that have to be investigated during the formulation studies. Especially for poorly soluble drugs, the composition of drug-loaded fluid should avoid the drug sedimentation which is the main cause of dose inaccuracy but should maintain the viscosity in a range acceptable for printing [108]. Moreover, several parameters (e.g., evaporation time, droplet size formation) have to be set-up to print homogenously the drug onto the subtract [117].

3D printing technologies based on material extrusion are also widely studied for tailoring the drug product according to the patients’ needs. Based on the preparation technique used, the extruded materials can be solid (e.g., FDM, HMRE) or semisolid (e.g., SSE). Moreover, the extruded material can be pre-formed filaments (e.g., FDM) or extemporaneously prepared (e.g., HMRE, SSE). Unlike binder jetting technologies, material extrusion ones do not require a preformed film onto the drug is deposed but the extruded material is directly printed in the designed dose and geometry. In particular, the shape of the dosage forms can be easily modified to improve the patient acceptability [118]. As a consequence, a future application of 3D printing technologies to the clinical practice will be advantageous to tailor both the drug dose and the morphological properties of the dosage form according to the patient needs, improving therapy adherence. In addition, the biopharmaceutical properties of the printed product can be custom-made by changing the formulation composition. Different kinds of pharmaceutical-grade polymers have already been tested for FDM 3D printing applications [119]. The design of multi-compartment devices, able not only to delivery FDCs [112,113], but also to control their release kinetics to maximize the drug bioavailability, can be achieved [120]. However, although FDM technology is very promising in an industrial-scale, its application to pharmacy setting or at patient home is limited by the unavailability on the market of drug-loaded filaments to prepare the medicinal product and tailor the dose strength. In this context, SSE and HMRE technologies seem more versatile and do not require high temperature to complete the printing process [107]. As well as FDM 3D printing, SSE and HMRE technologies permit to produce fixed combination and to control of drug release kinetics modulating the composition and the geometry of extruded materials [107]. However, no pre-formed filaments are needed since the extruded material is a paste for SSE 3D printing [113] and a solid mixture of plasticized low-melting-point components for HMRE 3D printing [109]. For both technologies, the preparation method consists in the mixing of the API and other components with a mortar and pestle, in the loading in the extruder and in the printing of the product in the designed geometry. In the case of SSE technology, a solvent evaporation step is also required after the printing to eliminate all the residue of aqueous or organic solvents used during the preparation process. Considering that HMRE 3D printing does not require liquids in the preparation process, this technology is also promising to print single dosage forms directly on the packaging material, which further simplifies the preparation process for a pharmacy setting. Although very promising and in continuous improvement, regulatory concerns remain a major issue in the roll-out of 3D technology in manufacturing pharmaceutical products. Indeed, unlike medical devices for which regulatory agencies have started to release guidance, the production of medicinal products by 3D-printing is far to be properly regulated due to the novelty of this technological application. Only in 2015 did the FDA authorize the first industrial medicinal product (Spritam^®^) produced by 3D-printing. Although the current regulatory framework on industrial production/compounding of medicinal products is flexible enough to be applicable to 3D technologies, the regulatory agencies are still facing important challenges in the definition of proper standards based on the peculiar features of 3D-printed medicines to assure their quality throughout the manufacturing and distribution [121]. Regardless of these challenges, the application of 3D printing technologies is promising for the preparation of patient centric pharmaceutical drug product. On one side, the possibility to print FDCs may be advantageous to simplify the regimen of polypharmacy patients, such as the elderly [113]. On the other side, 3D-printing technologies permit to design the shape of the dosage forms according to the patient needs. For dermal medicinal products, it permits the medicine application area to be adapted to the skin surface [122]. Moreover, it could also be advantageous to prepare children-friendly shape (e.g., stars, flowers) to improve adherence in younger patients [112].

## 4. Conclusions

Several interventions have been proposed to increase medication adherence. When low adherence is ascribable to the treatment, patient centric drug product pharmaceutical design can help by improving the acceptability of the medicinal product. In this context, the design of the drug product offers the possibility to meet the needs and preferences of patients by the selection of the most appropriate dosage forms and formulation composition based on the peculiarities of the target patient population. Fixed dose combination products are greatly studied regarding promotion of adherence, since they can ensure schedule simplification due to reduced dosing frequency and pill burden. Moreover, novel technologies including 3D printing bring exciting opportunities for the preparation of personalized medicines and could play a paramount role in the near future. Even if PCDPD may not completely resolve complex issues contributing to the problem of nonadherence, it is a promising idea to be taken into account in a global strategy to promote adherence to medication. 

## Figures and Tables

**Table 1:** Patient-related characteristics relevant for patient centric pharmaceutical drug product design (adapted from References [30,32]).

Patient-Related Characteristics	Examples
Age	Organ and body functions, socioemotional status
Visual impairment	Blindness
Motoric impairment	Arm mobility, difficulty walking, manual dexterity
Swallowing impairment	Dysphagia
Cognitive impairment	Memory loss, dementia
Poor hand sensitivity	Control of movement and strength
Loss of hearing	
Dentition	
Health literacy	
Psychological distress	Negative perception, depressive disorders
Disease state	Comorbidities, disease disability
PK/PD	Renal and hepatic clearance
Psycho-social issues	Way of living, Employment status, access to caregivers

**Table 2:** Product-related characteristics relevant for patient centric pharmaceutical drug product design (adapted from References [30,32]).

Product-Related Characteristics	Examples
Route of administration	Oral, inhalation, rectal, vaginal, dermal, parenteral
Product strength concentration	
Type of dosage form	Tablet, oral solution, ointment
Site of dermal application	Arm, feet, back
Appearance	Product size, shape, colour, embossing
Swallowability	Related to tablet size, shape, coating/waxing, liquid viscosity, mouth feel, Taste
Dose to therapeutic effect	Number of tablets, total volume of liquid
Dosing regimen	Dosing frequency, duration of treatment
Packaging	Inner/outer, labelling
Container closure system	
Dosing and administration devices	Syringes, applicator
Any handlings to be conducted prior to use	Opening capsules, measuring liquids, mixing
Instructions for use	Complexity
Caregiver assistance	Injections

**Table 3:** Fixed dose combination for the treatment of chronic conditions.

Condition	Fixed Dose Combinations	Year of Marketing Authorization
Angina	Beta-blocker/HCN Channel blocker	2015
COPD	LABA/LAMA	2013
	ICS/LABA	2013
	ICS/LABA/LAMA	2017
Dyslipidaemia/Atherosclerosis	Statin/Cholesterol absorption inhibitor	2004
	Statin/Niacin	2008
	Statin/Aspirin	2004
	DP1 anti-flushing/Niacin	2008
Heart failure	Beta-blocker/ACEI	2015
	Beta-blocker/HCN Channel blocker	2015
	ARB/Diuretic	1998
	ACEI/Diuretic	1997
HIV	NRTI/NRTI	1998
	PI/PI	2001
	NRTI/NRTI/NRTI	2000
	NRTI/NRTI/NNRTI	2007
	NRTI/NRTI/Integrase inhibitor/Booster of integrase inhibitor	2013
Hypertension	ACEI/CCB	2008
	ACEI/Diuretic	1997
	ACEI/Beta-blocker	2015
	ARB/CCB	2007
	ARB/Diuretic	1998
	CCB/Diuretic	2013
	ARB/CCB/Diuretic	2009
	ACEI/CCB/Diuretic	2014
	ACEI/CCB/Statin	2015
	ARB/Diuretic/CCB/Beta-blocker	-
Osteoporosis	Bisphosphonates/Cholecalciferol	2005
Post myocardial infarction	Aspirin/Thienopyridines	2010
	Beta-blocker/ACEI	2015
Type II diabetes	Biguanide/Sulfonylurea	2016
	Biguanide/Glitazon	2003
	Sulfonylurea/Glitazon	2006
	Biguanide/DPP-4 Inhibitor	2007
	Glitazon/DPP-4 Inhibitor	2013
	Biguanide/Glinid	2008
	Biguanide/Glifozin	2014
	DPP-4 Inhibitor/Glifozin	2016

ACEI = angiotensin converting enzyme inhibitors; ARB = angiotensin receptor blockers; CCB = calcium channel blockers; COPD = chronic obstructive pulmonary disease; DPP-4 = dipeptidyl peptidase-4; HIV = human immunodeficiency virus; ICS = inhaled corticosteroid; LABA = long-acting beta2 agonist; LAMA = long-acting muscarinic antagonist; NNRTI = non-nucleoside reverse transcriptase inhibitor; NRTI = nucleoside reverse transcriptase inhibitor; PI = protease inhibitor.
